# A facile way to produce epoxy nanocomposites having excellent thermal conductivity with low contents of reduced graphene oxide

**DOI:** 10.1007/s10853-017-0969-x

**Published:** 2017-03-13

**Authors:** Ganiu B. Olowojoba, Sotirios Kopsidas, Salvador Eslava, Eduardo S. Gutierrez, Anthony J. Kinloch, Cecilia Mattevi, Victoria G. Rocha, Ambrose C. Taylor

**Affiliations:** 1 Mechanics of Materials Division, Department of Mechanical Engineering, Imperial College London, London, SW7 2AZ UK; 2 Centre for Advanced Structural Ceramics, Department of Materials, Imperial College London, London, SW7 2AZ UK; 3 Department of Chemical Engineering, University of Bath, Bath, BA2 7AY UK; 4 School of Engineering, Cardiff University, Cardiff, CF24 3AA UK

**Keywords:** Epoxy, Graphene Oxide, Dynamic Mechanical Thermal Analysis, Reduce Graphene Oxide, Epoxy Polymer

## Abstract

A well-dispersed phase of exfoliated graphene oxide (GO) nanosheets was initially prepared in water. This was concentrated by centrifugation and was mixed with a liquid epoxy resin. The remaining water was removed by evaporation, leaving a GO dispersion in epoxy resin. A stoichiometric amount of an anhydride curing agent was added to this epoxy-resin mixture containing the GO nanosheets, which was then cured at 90 °C for 1 h followed by 160 °C for 2 h. A second thermal treatment step of 200 °C for 30 min was then undertaken to reduce further the GO in situ in the epoxy nanocomposite. An examination of the morphology of such nanocomposites containing reduced graphene oxide (rGO) revealed that a very good dispersion of rGO was achieved throughout the epoxy polymer. Various thermal and mechanical properties of the epoxy nanocomposites were measured, and the most noteworthy finding was a remarkable increase in the thermal conductivity when relatively very low contents of rGO were present. For example, a value of 0.25 W/mK was measured at 30 °C for the nanocomposite with merely 0.06 weight percentage (wt%) of rGO present, which represents an increase of ~40% compared with that of the unmodified epoxy polymer. This value represents one of the largest increases in the thermal conductivity per wt% of added rGO yet reported. These observations have been attributed to the excellent dispersion of rGO achieved in these nanocomposites made via this facile production method. The present results show that it is now possible to tune the properties of an epoxy polymer with a simple and viable method of GO addition.

## Introduction

The starting material for the production of graphene-based polymer nanocomposites is frequently graphene oxide (GO), which is usually prepared either via an electrochemical method [1] or a chemical oxidation of graphite [2]. The latter method is often referred to as the Hummers method, or a variant of this process termed the modified Hummers method [3, 4]. These very popular preparation routes lead to an aqueous dispersion of GO, with the GO containing many oxygen-containing functional groups (OCFGs) such as carboxyl, epoxide, carbonyl and hydroxyl groups. The attachment of the OCFGs during the chemical oxidation changes the hybridization of the carbon atoms in the graphitic lattice from sp^2^ to sp^3^. This disrupts electron and phonon transport, leading to poor electrical and thermal properties, respectively, of the GO so prepared, although it has the potential to increase the ease of dispersion of the GO when used as a filler in the production of polymer nanocomposites. The application of the graphene-based polymer nanocomposites requires that the OCFGs are reduced, to give reduced graphene oxide (rGO), in order to partially or fully obtain the excellent properties of graphene. Various reduction strategies for the GO are well known and have been reported in the literature [5–7]. For example, before the polymer nanocomposite is produced, the GO in the aqueous dispersion may be chemically reduced, using hazardous reagents such as hydrazine. Alternatively, the GO when dispersed in particulate form in the aqueous medium may then be obtained in a solid form, via filtration or freeze-drying, before it is reduced and then dispersed in the chosen matrix by sonication or shear mixing, or a combination thereof [8, 9]. However, more recently, in situ reduction of the GO in the nanocomposite has gained in popularity [10–14]. This has arisen for several reasons. Firstly, if a good dispersion state of the GO exists in the aqueous medium it may be largely preserved, thereby circumventing the re-agglomeration which usually accompanies the reduction of the GO to rGO via the above-mentioned routes. Secondly, the in situ reduction route eliminates the extra processing step needed to reduce the GO prior to incorporation in a matrix. Thirdly, obtaining a well-dispersed phase of solid rGO in the polymeric matrix via sonication or shear mixing is far from easy. Therefore, the starting point of an aqueous dispersion of GO which is, as the final step, reduced to rGO in situ in the polymeric matrix offers a facile method for the production of such materials based upon an epoxy polymeric matrix.

Considering the work reported in the literature relevant to such a facile production method, Yang et al. [10] investigated GO/epoxy polymer nanocomposites produced by transferring the graphene oxide from an aqueous suspension into the epoxy resin via a two-phase extraction and then curing the well-dispersed GO/epoxy resin suspension. They reported significant improvements in the toughness and compressive failure strength (i.e. of 1185 and 48%, respectively) of the epoxy nanocomposites containing as little as 0.038 wt% of GO. However, the relatively low reduction temperature of 150 °C that they adopted is likely to have left significant quantities of OCFGs on the GO and hence give a poor thermal conductivity after this relatively limited thermal treatment for the epoxy polymer nanocomposite. Peng et al. [15] adopted a similar approach to produce rGO/epoxy polymer nanocomposites with an excellent dispersion of rGO nanosheets. The reduction of the GO was carried out when the GO was suspended in the epoxy resin (triglycidyl para-aminophenol) at 200 °C for 5 min, before curing the epoxy resin by adding 3,5-dimethylthio-2,4-toluenediamine.

In this paper, we present a facile and effective route to prepare graphene–epoxy polymer nanocomposites with a remarkable increase in the thermal conductivity of the epoxy polymer at relatively very low contents of rGO. The ability of the OCFGs to impart a high degree of compatibility of the GO with epoxy resin is first exploited by mixing an aqueous dispersion of GO with the epoxy resin. Next, the remaining water is removed by evaporation, leaving a GO dispersion in the epoxy resin, which is cured with an anhydride curing agent. The final step is to reduce the GO in situ in the epoxy polymeric matrix at 200 °C. The result is a good dispersion of rGO in the crosslinked epoxy polymer. The advantages of this facile production route, compared to previously reported routes, include: (a) the relatively high content of GO in the aqueous suspension used means that the processing time is considerably shorter; (b) it does not involve the use of hazardous GO reducing agents such as hydrazine; (c) in situ reduction of the GO after curing of the epoxy polymer eliminates the extra processing step of reducing the GO prior to incorporation into the matrix; (d) the difficulties associated with dispersing powdery rGO into the epoxy resin are avoided; and (e) the relatively high temperature of 200 °C used to reduce the GO ensures an effective in situ reduction of the GO without degrading the chosen epoxy polymer. The thermal and mechanical properties of the epoxy nanocomposites so produced are reported in the present paper, together with the remarkable thermal conductivities at relatively very low contents of rGO.

## Experimental

### Materials

A standard diglycidyl ether of bis-phenol A (DGEBA) liquid epoxy resin (Araldite LY556; Huntsman, UK) having an epoxide equivalent weight (EEW) of 185 g/eq. was used. The curing agent was an accelerated methylhexahydrophthalic acid anhydride (Albidur HE600; Evonik, Germany) having an anhydride equivalent weight (AEW) of 170 g/eq.

### Synthesis of graphene oxide

Graphene oxide was prepared via a modified Tour et al. [4] synthesis in a custom-built rig designed to employ up to 10 L of concentrated acid in two jacketed reactors with overhead stirrers. In a typical synthesis, a 10:1 mixture of concentrated acids (3 L H_2_SO_4_:0.3 L H_3_PO_4_) was added to 24 g of natural graphite flakes (150–500 µm; Sigma-Aldrich, UK) under vigorous stirring, followed by the addition of 144 g of KMnO_4_ (6 weight equivalent) in small portions. The reaction mixture was then kept at 50 °C under vigorous stirring for 18 h. The mixture was cooled to room temperature, and the oxidation reactions were stopped by a drop-wise addition of 1.72 L of 2 wt% aqueous H_2_O_2_. The GO suspension was washed by repeated centrifugation and re-dispersion in distilled water, using a Sorvall LYNX 6000 Superspeed Centrifuge (Thermo Scientific, UK). This washing procedure was repeated until the pH of the supernatant matched that of the used distilled water, which typically occurred after 16 washing cycles. Typically two low-speed (<1000 rpm) centrifugation cycles were then performed to remove un-exfoliated graphite particles.

### Preparation of the epoxy polymer nanocomposites containing rGO

**Figure 1:**
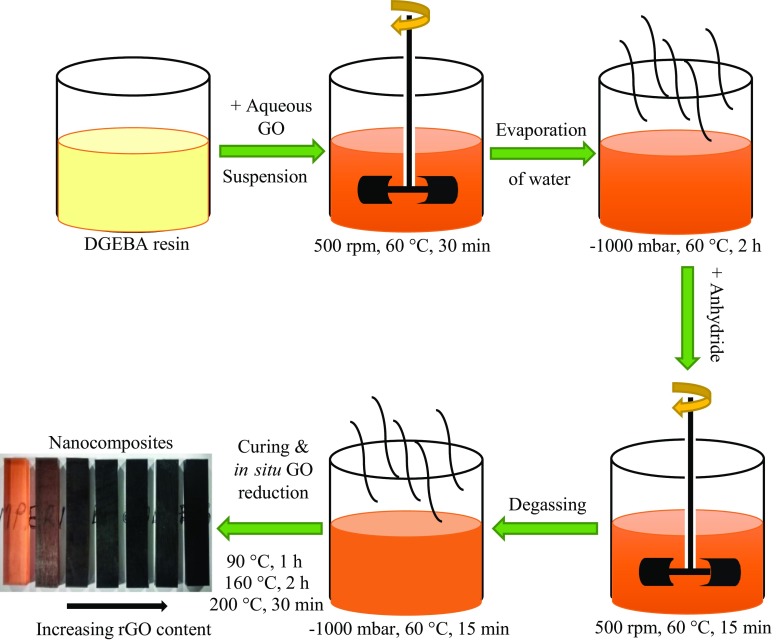
Schematic of the nanocomposite production route. The GO is thermally reduced in situ after the curing of the epoxy polymer nanocomposite. *The bottom left-hand* photograph shows the cured nanocomposites containing increasing contents of rGO from *left* to *right* (0–0.06 wt%)

A concentrated aqueous suspension of GO (13 mg/mL) was mixed with the epoxy resin by stirring first manually and then mechanically at 500 rpm using a radial-flow impeller for 30 min, as depicted in Fig. 1. The temperature was maintained at 60 °C to reduce the viscosity of the epoxy resin. Although aqueous GO suspensions are well known to form liquid crystalline structures at high contents [16], these are easily broken up by the mechanical agitation to allow a good dispersion of the aqueous GO suspension in the epoxy resin, owing to the compatibility between GO and epoxy. The resulting mixture was placed under vacuum at a gauge pressure of −1000 mbar and 60 °C for 2 h in order to evaporate the water and to remove any air bubbles. A stoichiometric amount of the anhydride curing agent was added to the GO/epoxy mixture. The weight ratio of epoxy resin to anhydride curing agent was 185:170, as according to their equivalent weights. The resulting mixture was stirred at 500 rpm for 15 min at a temperature of 60 °C. The mixture was then degassed again at −1000 mbar and 60 °C for 15 min to remove air bubbles and any remaining water. The degassed mixture was poured into preheated rectangular steel moulds with internal dimensions of 150 × 80 × 3 mm^3^ and was cured at 90 °C for 1 h and then post-cured at 160 °C for 2 h to produce bulk nanocomposite plates. A further thermal treatment step of 200 °C for 30 min was then undertaken to ensure an effective in situ thermal reduction of the GO in the crosslinked epoxy polymer nanocomposite [12, 17]. The contents of rGO prepared in the epoxy polymer nanocomposites were between 0.01 and 0.06 wt%.

The unmodified epoxy polymer control was prepared in the same way as outlined above. However, in this case, the concentrated aqueous GO suspension was replaced with deionized water whose volume was equivalent to that of the aqueous rGO suspension used for the preparation of the nanocomposite with 0.06 wt% rGO (i.e. the highest rGO content used).

### Characterization

#### Microscopy studies

Field-emission gun scanning electron microscopy (FEG-SEM) was used to image the dispersion of the rGO by examination of fractured samples of the nanocomposites. A Leo 1525 (Carl Zeiss, Germany) microscope was used, with an accelerating voltage of 5 kV. The samples were sputter-coated with a thin film of chromium prior to examination. Transmission electron microscopy (TEM) was carried out using a 2000FX microscope (JEOL, USA) employing an accelerating voltage of 200 kV. Thin slices (about 70 nm in thickness) were cut for TEM using a PowerTome XL ultramicrotome (RMC Products, UK). Atomic force microscopy (AFM) was carried out to determine the morphology of the GO using a MultiMode scanning probe microscope (Veeco, UK). The microscope was equipped with a NanoScope IV controller and an E scanner. The concentrated aqueous GO suspension (13 mg/mL) was diluted to 0.1 mg/mL using deionized water. The diluted GO suspension was then dip-coated onto a mica sheet (Agar Scientific, UK) and allowed to dry. Height and phase images were captured by AFM at a resolution of 512 pixels × 512 pixels and a scan speed of 1 Hz, using silicon probes in a tapping mode of operation.

#### Infrared spectroscopy studies

Fourier transform infrared spectroscopy (FTIR) was carried out on freeze-dried GO and thermally-reduced GO samples using a Spectrum 100 FTIR Spectrometer (PerkinElmer, UK). Aqueous GO suspensions, produced as outlined above, were freeze-dried using a Powerdry LL1500 freeze dryer (Thermo Scientific, UK) to obtain the dry GO powder used for FTIR and TGA. The freeze-dried GO was thermally reduced according to the cure plus reduction schedule adopted for the production of the nanocomposites (i.e. 90 °C for 1 h, 160 °C for 2 h and 200 °C for 30 min).

#### Mechanical and thermo-mechanical studies

Uniaxial tensile tests were conducted at room temperature using a universal testing machine (5584; Instron, UK). Dumbbell specimens having a gauge length of 25 mm were machined from 75 × 13.5 × 3 mm^3^ pieces cut from the bulk nanocomposite plates. A displacement rate of 1 mm/min was used, and the strain was measured using a clip-on extensometer (2620-601; Instron, UK). Tensile modulus values were calculated between strains of 0.05 and 0.25%. The tensile properties were averaged from the results obtained from a minimum of five specimens.

The thermo-mechanical properties of the nanocomposites were determined by dynamic mechanical thermal analysis (DMTA) using a Q800 analyser (TA Instruments, UK). Rectangular samples with dimensions of 60 × 10 × 3 mm^3^ were cut from the bulk nanocomposite plates. The samples were subjected to a temperature sweep from 30 to 200 °C at a heating rate of 2 °C/min in dual-cantilever mode at a frequency of 1 Hz using an oscillation strain of 0.05%. The glass transition temperature, *T*_g_, of each sample was taken at the maximum of the tan *δ* curve. The number average molecular weight between crosslinks, *M*_nc_, was calculated from:

1 $$ M_{\text{nc}} = \frac{q\rho RT}{{E_{\text{r}} }} $$

where *q* is the front factor, *ρ* is the density of the epoxy determined at room temperature (1.2 g/cm^3^ [18]), *R* is the universal gas constant (8.314 J/kg K), *T* is the temperature and *E*_r_ is the rubbery storage modulus determined at a temperature of *T* = 453 K (180 °C). Since the density of the epoxy was determined at room temperature, Pearson and Yee [18] suggest a front factor, *q*, of 0.725.

#### X-ray studies

X-ray photoelectron spectroscopy (XPS) analysis of the GO and rGO was performed using a Theta Probe spectrometer (ThermoFisher Scientific, UK), operated at a base pressure of 1 × 10^−9^ mbar. The spectra were acquired using a MXR1 monochromated Al Kα X-ray source (*hυ* = 1486.6 eV). An X-ray spot of ~400 μm radius was employed. High-resolution, core-level C1s spectra were acquired using a pass energy of 20 eV. The GO sample spectra were charge-referenced against the C1s peak at 284.4 eV to correct for charging effects during acquisition. Quantitative surface chemical analyses were calculated from the high-resolution, core-level spectra following the removal of a nonlinear (Shirley) background. The manufacturer’s Avantage software was used which incorporates the appropriate sensitivity factors and corrects for the electron energy analyser transmission function.

X-ray diffraction (XRD) patterns of the samples were acquired using an X’Pert PRO diffractometer (PANalytical, UK). The diffractometer was equipped with a Cu Kα radiation source. A generator voltage of 40 kV and tube current of 40 mA were used for all measurements over a 2*θ* range of 5°–60°, using a step size of 0.02° and 20 s per step.

#### Thermal studies

Thermogravimetric analysis (TGA) was carried out on the nanocomposites and freeze-dried GO samples using a TGA/DSC 1 (Mettler Toledo, UK). The samples were analysed using a heating rate of 10 °C/min over a temperature range of 30–800 °C in either an air or a nitrogen atmosphere.

The thermal conductivities of the nanocomposites were determined by the laser flash technique using an LFA-427 Nanoflash (NETZSCH, Germany). The samples were coated with a thin layer of graphite prior to testing to increase absorption and transmission of infrared radiation energy. Three shots each were made on 10 mm × 10 mm samples, 2 mm thick, over a temperature range of 30–60 at 10 °C intervals. A laser voltage of 450 V and a pulse width of 0.8 ms were used. The thermal diffusivity of each sample was measured and converted to the thermal conductivity using the specific heat capacity estimated from that of a graphite control sample whose thermal diffusivity had been determined using the same set of conditions.

## Results and discussion

### Dispersion quality of GO in the aqueous suspension

**Figure 2:**
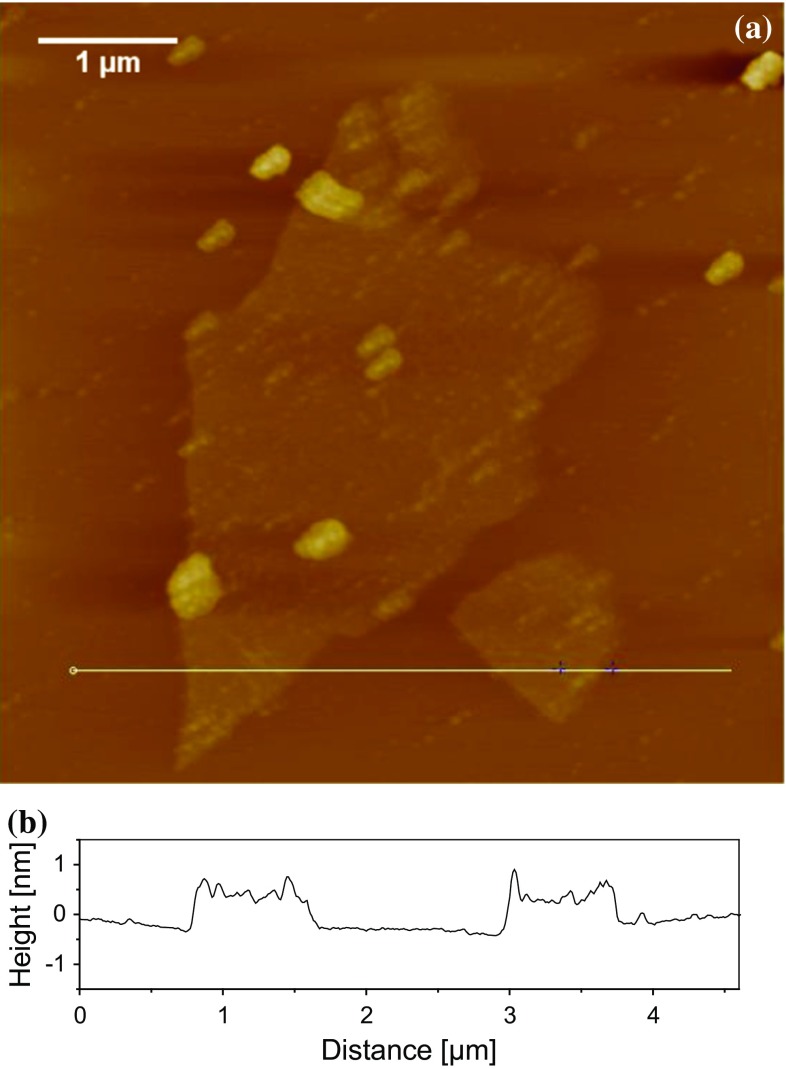
**a** AFM height image of GO sheets dip-coated onto a mica sheet from an aqueous solution of 0.1 mg/mL and **b** height profile along line shown in (**a**)

Preparation of the aqueous suspension of GO via the modified Hummers method leads to intercalation of OCFGs between the GO sheets formed during the oxidation of the graphite. This leads to an increase in the interlayer separation and hence a weakening of the attractive forces between the GO sheets. Moreover, the presence of the OCFGs imparts a high degree of compatibility of the GO with water and subsequently with the epoxy resin. These factors make it relatively easy to exfoliate and disperse the GO, with a minimum energy input, in the aqueous suspension. To illustrate this, a concentrated aqueous GO suspension of a content of 13 mg/mL was diluted to 0.1 mg/mL by adding deionized water, and the resulting suspension was vigorously shaken. The GO suspension was then dip-coated onto a mica sheet, dried and examined using AFM. Figure 2a shows the height image obtained, and Fig. 2b shows the height profile measured along the line indicated in Fig. 2a. A mean height of ~0.9 nm was measured for the GO deposited on the mica substrate. Now, a GO sheet has been reported as having a thickness in the range of 0.5–1.1 nm [5]. Thus, the measured thickness of the deposited GO indicates the occurrence of isolated individual GO sheets in the dilute aqueous suspension. This clearly demonstrates the ease with which GO can be readily exfoliated and dispersed with a minimal energy input.

### Selection of temperature for the reduction of graphene oxide

**Figure 3:**
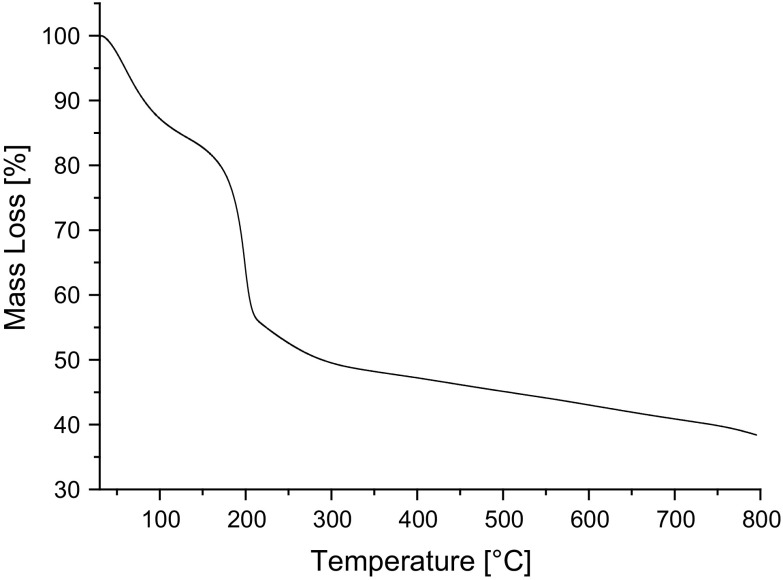
Thermogram of GO in a nitrogen atmosphere

Reduction of the GO was carried out in situ in the cured epoxy polymer; however, to establish suitable reduction conditions, it was necessary to carry out preliminary tests on freeze-dried (powdery) GO. TGA was performed on this powdery GO in a nitrogen atmosphere, and the resulting thermogram is shown in Fig. 3. The 15% mass loss at 100 °C is due to the removal of adsorbed hydroxyls and the by-products of low-temperature reduction of the GO, e.g. O_2_ and H_2_O [19]. A further 30% mass loss at 200 °C can be attributed to the thermal decomposition of OCFGs on the surface of the GO. It has previously been shown that most of the OCFGs attached to the basal aromatic plane of a GO sheet (hydroxyl and epoxide groups) can be removed by thermal treatment at 200 °C [20], and this largely restores the thermal, electrical and optical properties to those of graphene. Tang et al. [12] demonstrated that simply heating GO to 200 °C removes most of the OCFGs on the aromatic plane of GO and that holding the GO at this temperature for longer only leads to a marginal further increase in the weight of OCFGs lost. However, the OCFGs which are attached to the edges of the GO sheets are more thermally stable and will only decompose at temperatures higher than 200 °C [5], as seen from the gradual mass loss in the temperature range from 300 to 800 °C, see Fig. 3. Although it would be useful to reduce the GO at temperatures higher than 200 °C, degradation of the epoxy polymer will occur. Avoiding such degradation limits the reduction temperature that can be used.

### Effect of thermal treatment time at 200 °C on the reduction of GO

**Figure 4:**
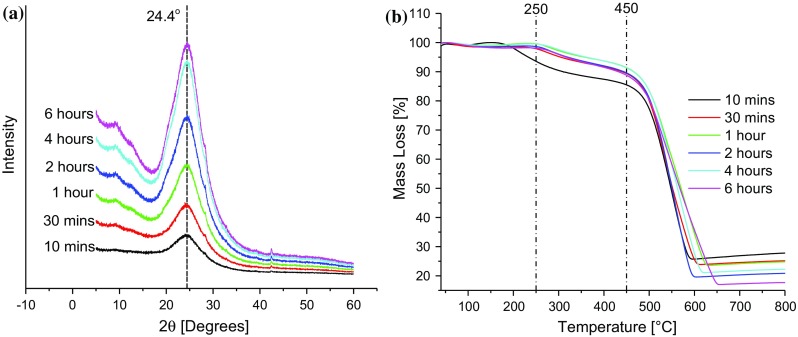
Effect of treatment time on the reduction of GO as studied by **a** XRD and **b** TGA

Once the reduction temperature of 200 °C was selected, the effect of the thermal treatment time on GO was investigated to allow a suitable reduction time to be chosen. Freeze-dried (powdery) GO samples were reduced at 200 °C for different lengths of time ranging from 10 min to 6 h, and Fig. 4a shows the resulting X-ray diffraction patterns of the rGO samples. The measured peak positions and interlayer spacings are shown in Table 1. All the rGO samples showed (002) diffraction peaks at ~24.4°, corresponding to an interlayer spacing of ~0.36 nm. No significant change was observed in the peak positions and interlayer spacings of the rGO samples, indicating that the amount of OCFGs removed by thermal dissociation does not correlate strongly with the reduction time of the GO.

**Table 1:** XRD, TGA and XPS data for rGO samples after thermal treatment at 200 °C for different periods of time

GO thermal treatment time (h)	XRD peak properties and interlayer spacing		TGA mass loss, 250–450 °C (%)	XPS abundance of functional groups (%)			
	Position (degrees)	d-spacing (nm)					
				C–C (284.7 eV)	C–OH (286.3 eV)	C=O (288.7 eV)	HO–C=O (289 eV)
0.17	24.42	0.364	8.01	73.45	20.41	2.83	3.30
0.5	24.47	0.364	8.50	69.97	21.48	3.51	5.04
1	24.57	0.362	8.31	69.47	20.53	4.68	5.32
2	24.46	0.364	8.93	72.64	17.40	3.44	6.53
4	24.57	0.362	8.30	73.65	18.06	3.24	5.06
6	25.03	0.356	9.20	72.79	19.95	2.85	4.41

**Figure 5:**
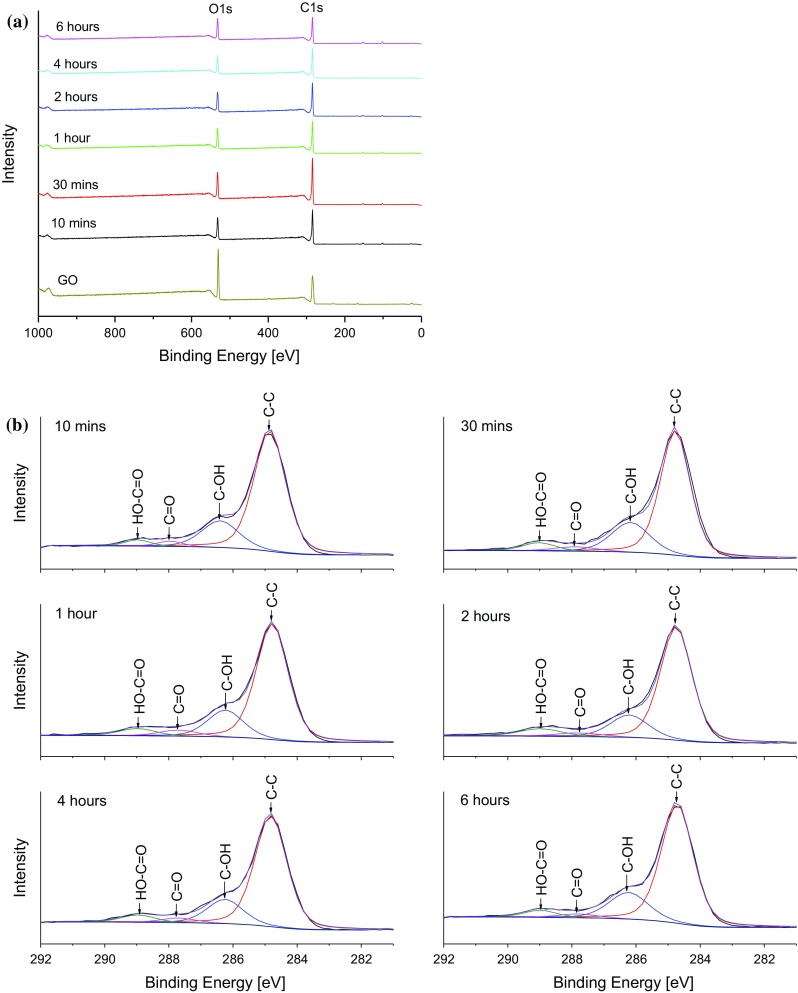
Effect of thermal treatment time on the reduction of GO as studied by XPS **a** survey spectra and **b** high-resolution C1s spectra

The thermograms obtained in air for the rGO samples are presented in Fig. 4b. The values of the mass loss over the temperature range of 250–450 °C are shown in Table 1, and this reduction can be attributed to the loss of OCFGs which are attached to the edges of the GO sheets. The mass loss in the temperature range of 450–650 °C can be attributed to the thermal oxidation of the graphene material. Table 1 also shows that the mass loss in the temperature range of 250–450 °C is largely insensitive to the GO thermal treatment time. The XPS spectra of the rGO samples are shown in Fig. 5a, while the corresponding high-resolution C1s spectra are shown in Fig. 5b. Peak deconvolution allows the quantification of the relative amounts of the different moieties bonded to the carbon atoms, as shown in Table 1.

There is little variation in the relative amounts of the carbon atoms bonded to other carbon atoms or to OCFGs as the thermal treatment time is increased. Although Jeong et al. [20] have previously indicated that the relative amount of OCFGs removed by thermal decomposition could be a function of the thermal treatment time, Tang et al. [12] concluded that the GO thermal treatment time has no effect. The results shown indicate that the GO thermal treatment time has little or no effect on the amount of OCFGs removed at 200 °C.

### Effect of the chosen reduction schedule for GO

**Figure 6:**
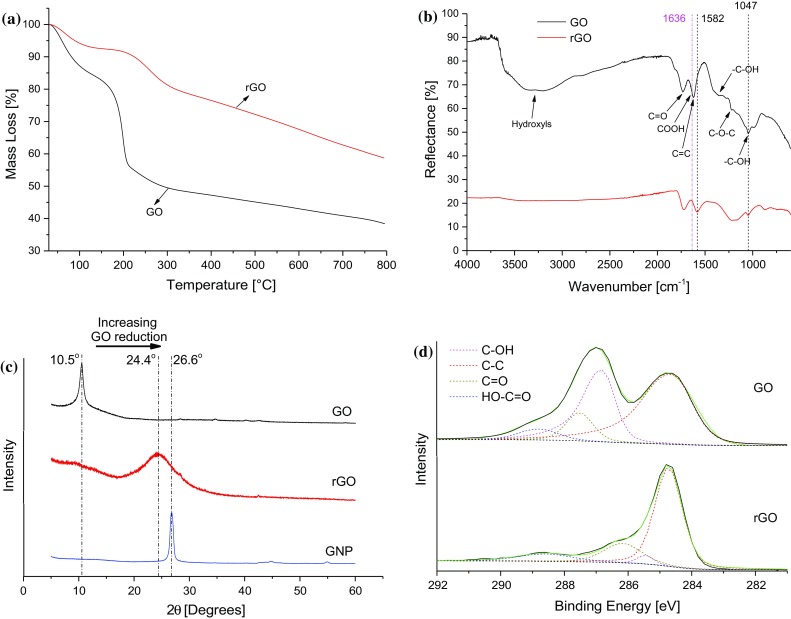
**a** Thermograms of GO and rGO in a nitrogen atmosphere, **b** FTIR spectra of GO and rGO, **c** XRD patterns of GO before and after reduction (the XRD pattern of a commercially-available GNP is also shown for comparison) and **d** XPS of GO before and after reduction, showing the decrease in the concentrations of OCFGs present after reduction

Based on the results of the above experiments, to reduce the GO without degrading the epoxy polymer, the reduction of the GO in the cured epoxy polymer nanocomposite was carried out in situ using a thermal treatment schedule of 200 °C for 30 min. Figure 6a compares the results of TGA in a nitrogen atmosphere of powdery GO before reduction with that of rGO reduced according to the complete cure and reduction schedule discussed above (i.e. 90 °C for 1 h, 160 °C for 2 h and 200 °C for 30 min). For the rGO, the 7% mass loss at 100 °C may be attributed to the removal of adsorbed hydroxyls and the by-products of low-temperature reduction of the GO, e.g. O_2_ and H_2_O [19], see Fig. 6a. Although the thermal treatment to form rGO removes most of the OCFGs on the aromatic plane of GO, which decompose at 200 °C, the OCFGs on the edges of the GO sheets are more thermally stable. These decompose at higher temperatures [5], giving rise to the gradual mass loss between 300 and 800 °C in Fig. 6a.

Figure 6b shows the FTIR spectra of the powdery GO and rGO. For GO, a broad peak corresponding to adsorbed hydroxyl groups from C–OH, HO–C=O and H–OH can be seen between 3146 and 3377 cm^−1^. The peaks at 1734, 1636, 1621.5 and 1223.5 cm^−1^ correspond to C=O, HO–C=O, C=C and C–O–C stretching, respectively, while those at 1351 and 1047 cm^−1^ correspond to C–OH stretching in the basal aromatic plane and at the edge of the GO, respectively [5, 21–23]. After reduction, several changes can be observed in the infrared absorption spectrum. Firstly, the peak at 1636 cm^−1^ corresponding to HO–C=O disappears, while the C–OH and C–O–C peaks originally at 1351 and 1223.5 cm^−1^, respectively, in GO are replaced by a broad peak of cyclic moieties in rGO. It can be seen, however, that the peak at 1047 cm^−1^ which corresponds to the stretching of C–OH attached to the edge of the GO sheet is unchanged. It is believed that these hydroxyl groups which are attached to the edge of the GO sheet are very stable and will only dissociate at temperatures in excess of 650 °C [5]. Tang et al. [12] obtained similar results. In addition to the removal of OCFGs during reduction, it can be seen that the C=C stretching peak is shifted from 1636 to 1582 cm^−1^. This is indicative of an increase in the recovery of sp^2^-hybridized carbon domains after the reduction of the GO.

The position of the (002) diffraction peak in XRD can usually be correlated to the size of the diffraction grating (in this case, the chemical heterogeneity of the GO largely determines the interlayer spacing). The reduction in chemical heterogeneity as the GO is reduced can be seen very clearly by the position of the diffraction peaks in the XRD patterns of GO and rGO shown in Fig. 6c. The XRD pattern of commercially-available thermally-reduced graphene nanoplatelets (GNPs) is shown for comparison. It can be seen that the (002) diffraction peak at 2*θ* = 26.6° in GNP is shifted to 2*θ* = 10.5° in GO after chemical oxidation. However, the (002) peak is partially restored to 2*θ* = 24.4° in rGO as many of the OCFGs are removed during thermal reduction. The corresponding interlayer spacing, as measured, decreases from 0.84 nm in GO to 0.36 nm in rGO as the OCFGs intercalated between individual sheets are removed [24–26]. By comparison, the measured interlayer spacing of pristine GNPs is 0.34 nm. The GO reduction process often leads to increased dislocations and imperfections in the graphitic lattice as some carbon atoms are lost in the form of CO_2_ or CO during reduction [5]. These lattice imperfections, as well as stacking faults generated in the restacking of graphitic sheets during reduction, may be contributing factors to the broadening of the (002) peak in rGO as observed in Fig. 6c [26].

Figure 6d shows the high-resolution C1s XPS spectra of GO and rGO, as well as the associated deconvoluted peaks which quantitatively represent the bonds between carbon atoms on the GO and other moieties. The relative abundance of the C=C bonds increased from 50.61% for GO to 69.97% for rGO. A decrease in the OCFGs bonded to carbon atoms was, however, observed; the C–OH groups decreased from 33.49% in GO to 21.48% in rGO, the C=O groups decreased from 10.19% in GO to 3.51% in rGO, and the HO–C=O groups decreased from 5.71% of GO to 5.04% in rGO. A significant reduction in the concentrations of the OCFGs bonded to carbon atoms can thus be observed upon reduction of GO, in accordance with the TGA, FTIR and XRD results previously discussed. These results clearly demonstrate the effectiveness of the GO reduction strategy adopted in the present work for the preparation of the rGO/epoxy nanocomposites.

### Dispersion of rGO in the nanocomposites

**Figure 7:**
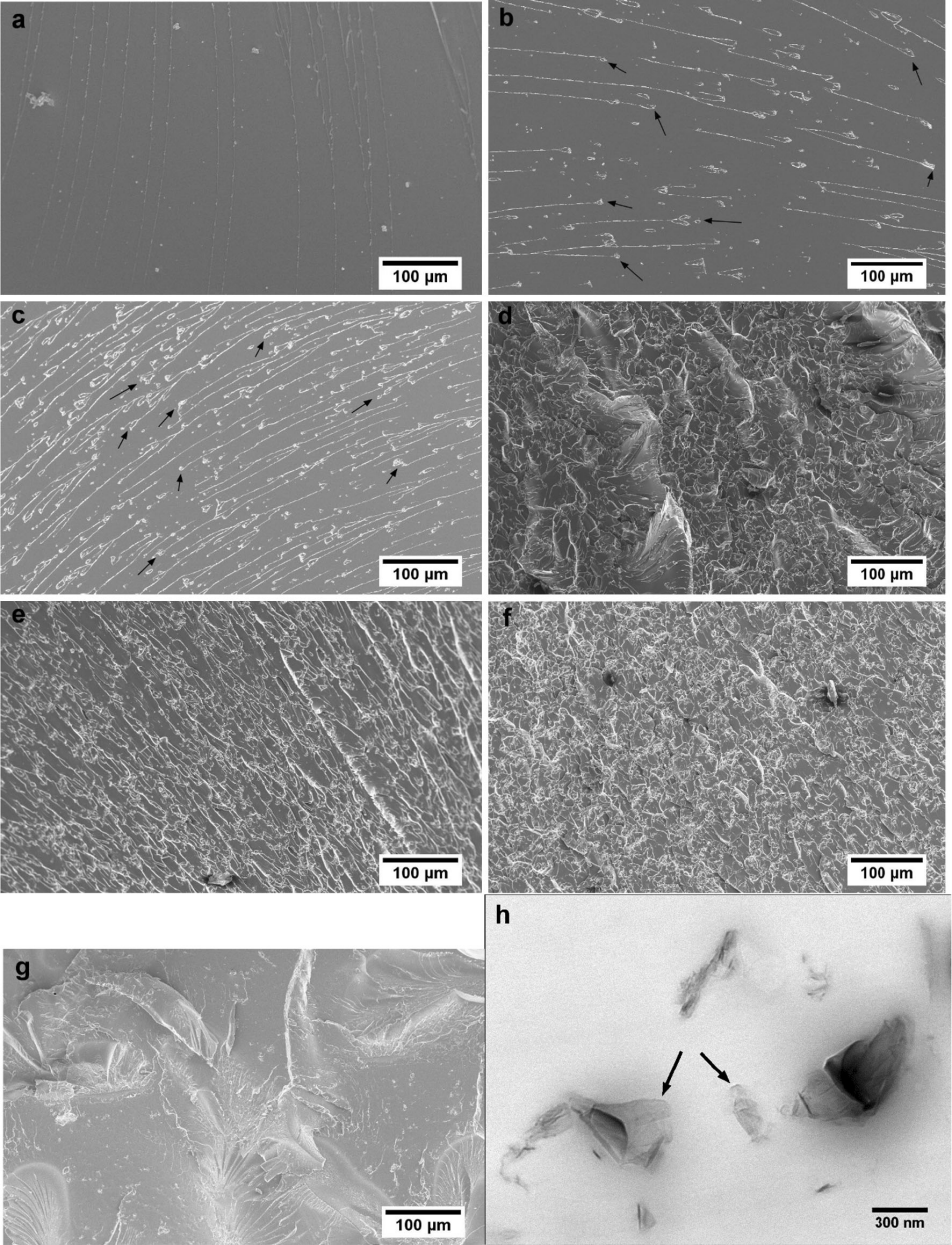
FEG-SEM of fractured surfaces of rGO/epoxy nanocomposites for **a** unmodified epoxy, **b** 0.01 wt%, **c** 0.02 wt%, **d** 0.03 wt%, **e** 0.04 wt%, **f** 0.05 wt%, **g** 0.06 wt% of rGO and **h** TEM of 0.06 wt% nanocomposite showing isolated sheets of rGO

For the rGO/epoxy polymer nanocomposites, where the GO is reduced in situ, it is instructive to first consider how the rGO is dispersed in the polymer matrix. The size and dispersion of the rGO sheets can be imaged using FEG-SEM on fractured samples. For convenience, the fracture surfaces of samples broken during tensile tests were used. (Note that the results of the tensile tests are discussed separately below). The unmodified epoxy is a homogeneous polymer as expected, see Fig. 7a. Note that the white lines in Fig. 7a are river lines caused by the brittle fracture of the polymer [27], and for the purposes of considering the dispersion of the rGO, such features associated with fracture can be disregarded in Fig. 7.

The images shown in Fig. 7b, c, d, e, f indicate a good dispersion of rGO in the epoxy for the nanocomposites containing 0.01–0.05 wt% of rGO, and significant agglomeration could not be detected even at high magnifications. Figure 7h shows a TEM image of the nanocomposite filled with 0.06 wt% of rGO. Isolated rGO sheets (indicated with black arrows) are clearly visible, which illustrate the good dispersion and relatively large flake size inherent in these nanocomposites. The infrared absorption spectra of GO, see Fig. 6b, show that both the surface and the edges of the GO are decorated with many OCFGs, among them epoxide groups. These OCFGs intercalated between the individual GO sheets lead to greater interlayer separation as measured for GO by XRD (0.84 nm, as opposed to 0.34 nm for GNPs, see Fig. 6c) and weaken both the van der Waals and π–π interactions between sheets [11]. The overall effect is to increase compatibility between the GO and the epoxy resin, as discussed below, and increase the ease of dispersion of the GO in the epoxy.

**Figure 8:**
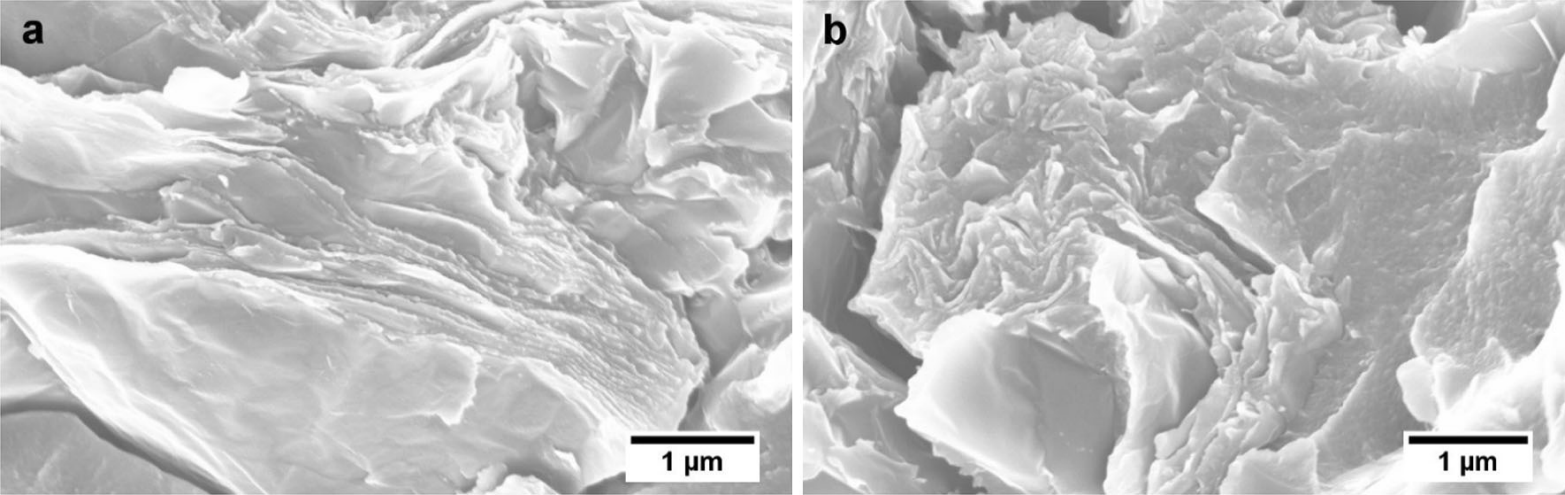
FEG-SEM images of 0.06 wt% rGO nanocomposite, showing self-assembly of rGO sheets

The microstructure of the nanocomposite containing 0.06 wt% rGO appears different to that of the lower contents, see Fig. 7g. Further examination at high magnifications revealed several rGO agglomerates, as shown in Fig. 8. The tendency of GO to self-assemble into liquid crystalline structures above a critical content in aqueous media, as well as in epoxy resins, has been reported [11, 16, 28–30]. Below the critical content, a more random arrangement of the GO is thermodynamically favoured, whereas at higher contents the steric hindrance between sheets and the excluded volume effect lead to self-assembly of the GO sheets into ordered structures. This may lead to agglomerated rGO nanoplatelets in the cured nanocomposites as seen in Fig. 8. This phenomenon may be exacerbated by a very good dispersion, as is the case in these nanocomposites, since the average statistical distance between the sheets is a minimum thereby increasing the attractive forces between them. These agglomerates could have an adverse impact on the mechanical properties of the nanocomposite, since they could act as stress contents. This is discussed in more detail in subsequent sections.

The morphology of the nanocomposites revealed a good dispersion of rGO. This has been attributed to the compatibility between the GO and the epoxy, as well as to the increased interlayer spacing between sheets due to the intercalated OCFGs which is expected to weaken the van der Waals and π–π interactions between individual sheets. Although increased agglomeration of rGO was observed in the nanocomposite with 0.06 wt%, isolated rGO sheets are visible which illustrate the good dispersion inherent in these nanocomposites.

### Thermo-mechanical properties of the nanocomposites

**Figure 9:**
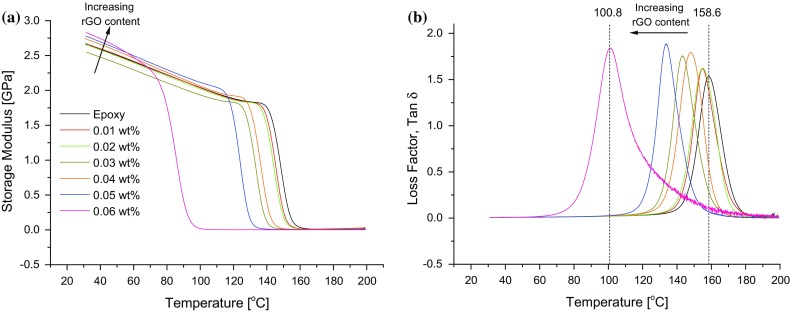
Thermo-mechanical properties of the rGO/epoxy polymer nanocomposites with varying rGO content showing **a** storage modulus and **b** tan *δ*

The thermo-mechanical properties of the nanocomposites were examined using dynamic mechanical thermal analysis (DMTA). Figure 9a, b shows the temperature dependence of the storage modulus (*E*′) and the damping coefficient (tan *δ*) for each of the nanocomposites. Selected values of the storage modulus plus the glass transition temperature and height of the tan *δ* peak are shown in Table 2. At 40 °C, the glassy storage modulus, *E*_g_′, increases from 2.59 GPa for the unmodified epoxy to 2.74 GPa for the nanocomposite with 0.06 wt% of rGO (i.e. an increase of 5.8%). In comparison, Chandrasekaran et al. [31] reported an *E*_g_′ increase of only 5.1% for a GNP/epoxy nanocomposite containing nearly double the filler content (i.e. 0.1 wt% of GNP), at 25 °C. The notable increase in *E*_g_′ observed in the present work is due to the high modulus of the rGO and the processing route adopted. The latter takes advantage of the relatively large flake size due to the minimal mechanical energy input during processing, the compatibility between the GO and the epoxy as well as the presence of intercalated OCFGs which lead to increased interlayer separation. These factors enable a good dispersion of the GO as seen in Fig. 7. Contributing factors to the increase in *E*_g_′, as reported in the literature, may be the wavy topology of rGO and the imperfections in the rGO lattice resulting from the sp^3^-hybridized domains which allow for better mechanical interlocking between the rGO and the polymer matrix [10]. Furthermore, the OCFGs react with the epoxy leading to the formation of covalent bonds, and therefore a better filler–matrix interface, and an increase in the load transfer characteristics is achieved, as exemplified by the observed increase in *E*_g_′ [10]. Indeed, the effective modulus of the rGO can be calculated using the rule of mixtures from the values of *E*_g_′ for the unmodified epoxy and the nanocomposite with 0.06 wt% of rGO. A value of 420 GPa is obtained for the effective modulus of the rGO, which compares well with literature values [32].

**Table 2:** Thermo-mechanical properties of rGO/epoxy nanocomposites as obtained by DMTA

rGO content (wt%)	Storage modulus (*E*_g_′) at 40 °C (GPa)	Storage modulus (*E*_r_′) at 180 °C (MPa)	Glass transition temperature (*T*_g_) (°C)	Height of tan *δ* peak	Molecular weight between crosslinks (*M*_nc_) (gmol^−1^)
0.00	2.59	11.5	158.6	1.53	285
0.01	2.60	10.1	154.9	1.61	324
0.02	2.58	9.8	154.2	1.62	334
0.03	2.48	7.6	143.0	1.76	431
0.04	2.66	8.2	148.1	1.79	399
0.05	2.70	3.7	133.5	1.87	886
0.06	2.74	1.6	100.8	1.84	2048

The glass transition temperature of the unmodified epoxy was measured to be 158.6 °C, see Table 2. The *T*_g_ of the nanocomposites decreases with increasing rGO content, for example, the *T*_g_ decreased by 57.8 °C (i.e. 57%) with 0.06 wt% of rGO, as shown in Fig. 9b. The maximum damping amplitude, as shown by the height of the tan *δ* peak, see Table 2, increases with increasing rGO content. These changes can generally be correlated with the crosslink density of the polymer [33]. The number average molecular weight, *M*_nc_, between crosslinks estimated from Eq. 1 for each nanocomposite is shown in Table 2. The value of *M*_nc_ clearly increases, so the epoxy matrix becomes less highly crosslinked, with increasing rGO content. This is attributed to the fact that in situ reduction of the GO leads to the opening and/or elimination of OCFGs which may lead to the formation of covalent bonds between the GO and the epoxy matrix as mentioned previously. Furthermore, GO has been reported to promote the homopolymerization of epoxy resin [13]. These factors may change the molecular weight between crosslinks of the epoxy during crosslinking, as has been reported previously [10, 11, 34, 35]. Indeed, nanoclays are known to produce a similar effect when used as fillers in epoxy polymers, e.g. [36, 37].

### Thermal stability of the nanocomposites

**Figure 10:**
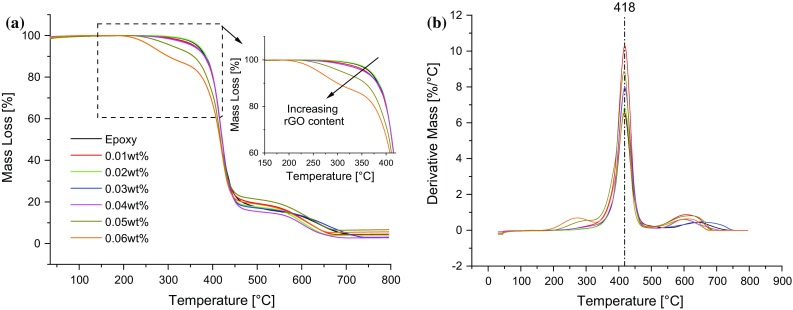
Thermal stability of the nanocomposites from TGA with varying rGO content, **a** mass loss and **b** derivative mass loss. The *inset* of **a** illustrates the decreasing thermal stability of the nanocomposites between 200 and 400 °C with increasing rGO content

The thermal stability of the nanocomposites in air was investigated by thermogravimetric analysis (TGA). Thermograms and derivative thermograms (DTGs) are shown in Fig. 10a, b, respectively. The thermogram of the unmodified epoxy is characterized by a two-step degradation process, see Fig. 10a. The mass loss between 350 and 450 °C can be attributed to the thermal oxidation of the epoxy, and that between 450 and 700 °C to the char formed from the thermal decomposition of the epoxy and to the rGO for the nanocomposites. Note that nothing happens below 200 °C as this was the temperature used for the thermal treatment. However, the nanocomposites are largely characterized by a three-step degradation behaviour. The extra step is the mass loss observed for the nanocomposites between 200 and 350 °C (inset of Fig. 7a), which increases with rGO content, especially at relatively high rGO contents. The mass loss observed for each of the nanocomposites up to 350 °C (*W*_350_) is shown in Table 3. A steady increase in *W*_350_ with increasing rGO content can be observed, except for the nanocomposite containing 0.02 wt% rGO. The onset temperature of thermal degradation, *T*_on_, also decreased with increasing rGO content from 287.4 °C for the unmodified epoxy to 207.1 °C for the 0.06 wt% nanocomposite (i.e. a decrease of 80.3 °C), in accordance with the trend in *T*_g_ shown in Fig. 9b. The inconsistencies observed in *T*_on_ and *W*_350_ for the 0.01 and 0.02 wt% rGO nanocomposites may be attributed to experimental variation. The maximum degradation temperature, *T*_max_, and the temperature of 50% mass loss, *T*_50%_, both remained largely unchanged, see Table 3. The changes observed in *T*_on_ and *W*_350_ are indicative of decreasing thermal stability with increasing rGO content, although increases in thermal stability with increasing rGO content have also been reported in the literature [11, 38].

**Table 3:** Thermal stability data from TGA for the rGO/epoxy nanocomposites

rGO content (wt%)	Onset temperature of thermal degradation (*T*_on_) (°C)	Mass loss at 350 °C (*W*_350_) (%)	Temperature of 50% mass loss (*T*_50%_) (°C)	Temperature of maximum degradation (*T*_max_) (°C)
0.00	287.4	2.69	420.7	416.9
0.01	270.6	3.80	420.7	418.8
0.02	284.7	2.29	420.7	418.0
0.03	257.4	4.36	421.7	418.3
0.04	253.8	4.38	421.7	418.5
0.05	238.9	8.86	417.3	416.7
0.06	207.1	14.48	416.4	418.3

As discussed earlier, some OCFGs remain attached to the rGO sheets after reduction, as shown in Fig. 6a, b, c, d. It therefore seems reasonable to expect an increase in the concentrations of undissociated OCFGs as the content of rGO increases. This can lead to increasing thermal instability, as shown in Fig. 10 and Table 3, since the OCFGs may thermally dissociate at high temperatures and oxidize the epoxy matrix.

The reaction between pendant OCFGs on the GO surface and epoxy resin is well known. Yang et al. [10] have shown that DGEBA molecules can be grafted onto GO by reaction with pendant OCFGs in an aqueous medium if a mixture of GO and epoxy resin is heated at 50 °C for 4 h. Wan et al. [39] prepared DGEBA-functionalized GO sheets by dispersing GO sheets in acetone via bath sonication in the presence of DGEBA resin at 70 °C using NaOH as the catalyst. They confirmed the grafting of DGEBA onto the GO sheets using a combination of XPS, XRD, AFM, TEM and Raman spectroscopy. The process adopted in this work for the removal of water (60 °C, −1000 mbar for 2 h) encourages the reaction of the OCFGs on the GO with the epoxide groups in the DGEBA. Galpaya et al. [13] have shown that the OCFGs on GO can also catalyse the homopolymerization of DGEBA. As mentioned earlier, these factors have the potential to change the crosslink density and hence could be expected to affect the thermal stability of the nanocomposites.

### Tensile properties of the nanocomposites

**Figure 11:**
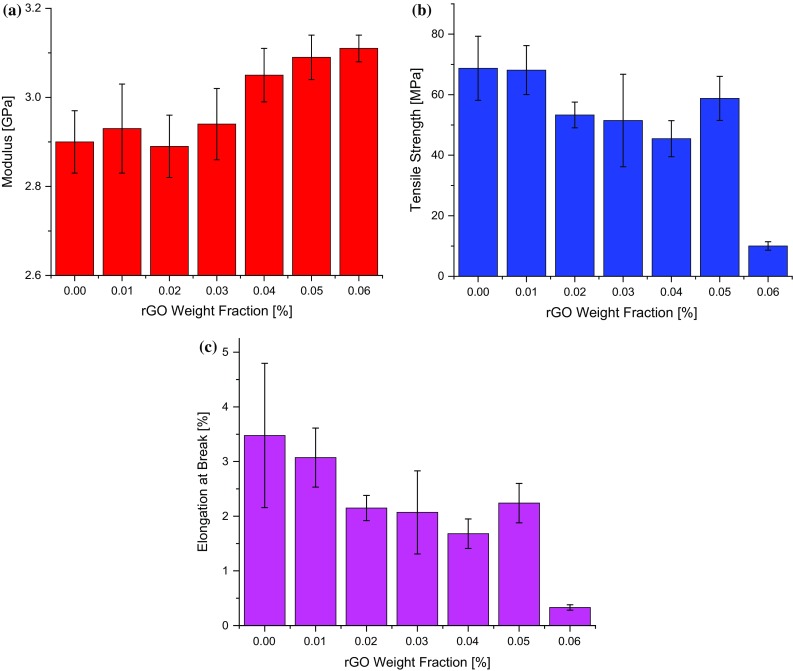
**a** Tensile modulus, **b** tensile strength and **c** elongation at break of the rGO/epoxy nanocomposites

Figure 11a shows the measured tensile modulus values for the nanocomposites. Although a slight decrease in modulus for the nanocomposite with 0.02 wt% rGO was observed, the modulus generally increased with increasing rGO content from 2.90 ± 0.07 GPa for the unmodified epoxy to 3.11 ± 0.03 GPa for the nanocomposite with 0.06 wt% rGO content (i.e. 7.2% increase). Wan et al. [39] reported a similar increase of about 7% in modulus upon the addition of 0.5 wt% of DGEBA-functionalized rGO to an epoxy matrix (i.e. nearly 10 times the weight of rGO used in this work), while Zaman et al. [34] observed a modest 3% increase in modulus on adding 1 wt% of 4,4′-methylene diphenyl diisocyanate (MDI)-functionalized GNP to epoxy. The effective modulus of the rGO can be calculated from the tensile modulus values using the rule of mixtures, as was done from the values of *E*_g_′, and a value of 580 GPa is obtained for the effective modulus of the rGO, which compares well with the 420 GPa calculated from the values of *E*_g_′ and with literature values [32]. The notable increase in modulus observed in this work may be attributed to the nanocomposite preparation route adopted, which provides for a good rGO dispersion and excellent interfacial properties between the rGO and the epoxy matrix.

The tensile strength and elongation at break of the unmodified epoxy and of the rGO/epoxy nanocomposites are shown in Fig. 11b, c, respectively. There is a decrease in tensile strength and elongation at break with increasing rGO content. The tensile strength decreased from 68.7 ± 10.5 MPa for the unmodified epoxy to 10.0 ± 1.4 MPa for the nanocomposite with an rGO content of 0.06 wt%. Similarly, a decrease in the elongation at break was observed from 3.5 ± 1.3% for the unmodified epoxy to 0.33 ± 0.05% for the nanocomposite with 0.06 wt% of rGO.

**Figure 12:**
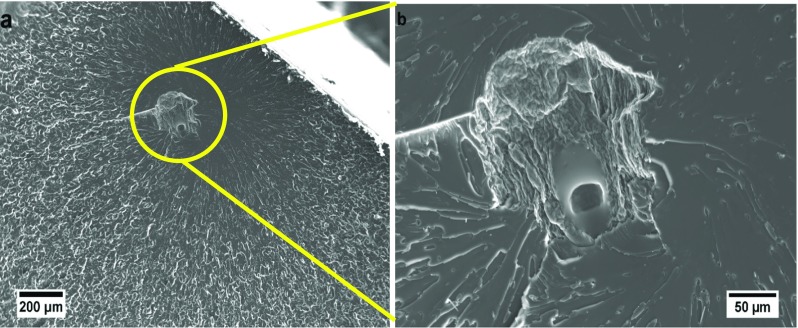
SEM of the fracture surfaces of the sample containing 0.02 wt% rGO after tensile testing, illustrating the defects present in the rGO/epoxy nanocomposites

Examination of the fractured surfaces of the tensile samples using FEG-SEM revealed defects in the nanocomposites, as shown in Fig. 12. These defects could arise from the gases (i.e. carbon dioxide, carbon monoxide and oxygen) released during the thermal dissociation of the OCFGs as the GO is reduced. Gases such as O_2_ and H_2_O, which are by-products of low-temperature reduction of the GO, can be released during the curing of the epoxy polymer [19] and thus could be trapped in the nanocomposites as bubbles by gelation. These bubbles would tend to act as stress concentrations, thus leading to a reduction in the tensile strength of the nanocomposites as observed in Fig. 11b. Although the interaction of rGO with the matrix may have led to strong interfacial bonds between the rGO and the polymer matrix, the reduction in crosslink density may have made the epoxy matrix more flexible, and the presence of such defects offsets these effects. Thus, the overall effect is a reduction in the tensile strength and the elongation at break of the nanocomposites, see Fig. 11. The sharp drop observed in the tensile strength between the nanocomposite with an rGO content of 0.05 wt% (58.8 ± 7.3 MPa) and that of 0.06 wt% (10.0 ± 1.4 MPa), is attributed to the increased rGO agglomeration in the 0.06 wt% nanocomposite, as shown in Fig. 8. These observations are consistent with the sharp reduction in *T*_g_ observed from 133.5 °C for the 0.05 wt% nanocomposite to 100.8 °C for the 0.06 wt% nanocomposite (Fig. 9b; Table 2). Although an increase in tensile strength for an in situ chemically-reduced GO/epoxy nanocomposite has recently been reported [11], other workers [34] have noted a reduction in tensile strength in the case of MDI-functionalized GNP/epoxy nanocomposites, a phenomenon which they ascribed to the reduction in crosslink density as a result of reaction between the MDI groups and the polymer matrix. The decrease in elongation at break with increasing rGO content observed in Fig. 11c may be attributed to the strong interfacial adhesion between the rGO and the matrix.

The fracture surfaces of the tensile samples were investigated using FEG-SEM, and the resulting micrographs are shown in Fig. 7. For the unmodified epoxy, Fig. 7a shows a relatively smooth surface with river lines characteristic of a brittle fracture. An increase in the surface roughness can be observed for the nanocomposites with 0.01 and 0.02 wt% of rGO (see Fig. 7b, c). Lines are still clearly visible on the surface, but these are not continuous, unlike for the unmodified epoxy. Instead they originate from the rGO sheets (as indicated by the arrows in Fig. 7b, c), resulting in tail-like features characteristic of crack deflection where the crack front has deviated when it encountered the particle. Such a toughening mechanism would be expected to result in enhanced fracture toughness, e.g. [40]. Chandrasekaran et al. [41] reported similar features in GNP/epoxy nanocomposites. The surface roughness increases further with increasing rGO content, as seen in Fig. 7d–f, corresponding to nanocomposites with rGO contents of 0.03–0.05 wt%. The agglomeration observed at 0.06 wt% of rGO results in a smoother fracture surface as there are fewer points to cause crack deflection because the rGO is agglomerated into fewer thicker particles, see Fig. 7g.

### Thermal conductivity

**Figure 13:**
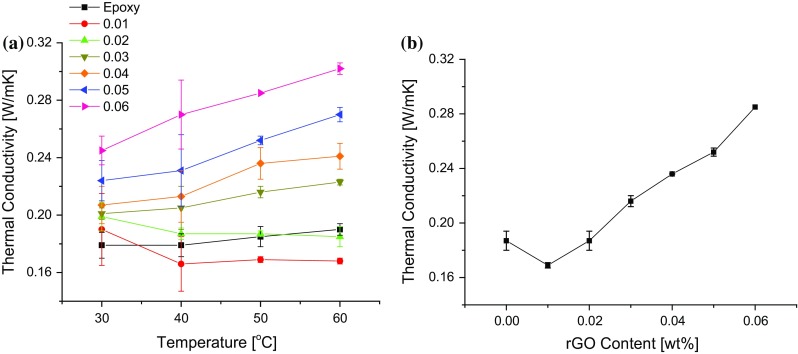
Thermal conductivity of unmodified epoxy and nanocomposites **a** at different temperatures and **b** at 50 °C

The thermal conductivities of the nanocomposites and the unmodified epoxy at different temperatures are shown in Fig. 13a. The thermal conductivity increases with increasing temperature and with increasing rGO content, except for the nanocomposite containing 0.01 wt% of rGO. At 30 °C, the thermal conductivity increases from 0.18 ± 0.009 W/mK for the unmodified epoxy to 0.25 ± 0.002 W/mK for the nanocomposite with 0.06 wt% of rGO (i.e. an increase of 39%). This trend is more clearly visible in the thermal conductivity of the nanocomposites measured at 50 °C, as shown in Fig. 13b, where an increase in thermal conductivity of 53% was measured for the 0.06 wt% nanocomposite (0.29 ± 0.001 W/mK compared with 0.19 ± 0.007 W/mK for the unmodified epoxy). The thermal conductivities of graphene and graphene-based hybrid nanocomposites are compared in Table 4 with that from the present work. Table 4 also shows the thermal conductivity enhancement factor, *κ*_e_, of each of the nanocomposites, which has been defined as the increase in thermal conductivity per unit mass of filler [42]. It can be seen that the *κ*_e_ value of 648 is by far the highest for the nanocomposites considered in Table 4. This shows that the thermal conductivity values observed in this work are among the highest ever reported for GNP/epoxy nanocomposites with such low contents of rGO. In the authors’ previous work [14], a thermal conductivity of 0.24 W/mK was reported for a 2 wt% rGO/epoxy nanocomposite, prepared by dispersing freeze-dried GO in epoxy via three-roll milling followed by in situ polymerization and reduction. Although this is a good increase in the thermal conductivity, the *κ*_e_ value of 16.7 is relatively small compared to that achieved in the present work, see Table 4.

**Table 4:** Comparison of the thermal conductivities of graphene-based/epoxy nanocomposites at room temperature

#	Filler	Content	Dispersion method	Thermal conductivity (W/mK)	Thermal conductivity increase (%)	Thermal conductivity enhancement factor^a^ (*κ*_e_)	Measurement method	Reference
1	rGO	0.06 wt%	Stirring	0.25	38.9	648	Laser flash	This work
2	GNP	20 wt%	Shear mixing	5.80	2800	140	Laser flash	[43]
3	MLG^b^	10.0 vol % (~16.9 wt%)	Sonication/centrifugation	5.10	2300	136	Laser flash	[44]
4	MLG^b^	2.8 vol % (5.0 wt%)	Sonication/stirring/shear mixing	1.50	650	130	Laser flash	[45]
5	rGO	3 wt%	Sonication/stirring	1.19	261	86.9	Hot disc	[46]
6	GNP	5.0 vol % (~8.8 wt%)	High shear mixing (non specific)	~1.45	621	70.6	Laser flash	[47]
7	rGO/nanosilica hybrid	1 wt%	Sonication/stirring	0.32	61.0	61.0	Laser flash	[48]
8	GNP	2.703 vol % (~4.8 wt%)	Sonication/stirring	~0.72	243	50.6	Laser flash	[49]
9	rGOx^c^	0.8 wt%	Sonication/stirring	~0.29	34.0	42.5	Laser flash	[50]
10	rGO	10.0 vol % (16.9 wt%)	Sonication/high shear mixing	1.26	556	32.9	Hot disc	[51]
11	rGO	15.8 wt%	Sonication/stirring	1.27	362	22.9	Laser flash	[52]
12	GNP	3 wt%	Three-roll milling	~0.37	68.2	22.7	Laser flash	[53]
13	rGO	2 wt%	Three-roll milling	0.24	33.3	16.7	Laser flash	[14]
14	GNP	2 wt%	Three-roll milling	~0.21	16.7	8.4	Laser flash	[31]
15	GNP	1.0 vol % (~1.8 wt%)	Stirring/sonication	0.23	10.0	5.6	Hot wire	[54]

^a^The thermal conductivity enhancement factor, *κ*_e_, is defined as the ratio of the percentage increase in thermal conductivity to the percentage loading by mass

^b^Multilayer graphene

^c^In situ reduced imidazole grafted GO

The thermal conductivity of particle-filled polymer composites depends on many factors: the intrinsic conductivity of filler and matrix, filler content, aspect ratio and dispersion, interfacial bonding between filler and matrix and thermal resistance offered by the interfacial layer [47]. For a highly-conductive filler such as graphene, a good dispersion will reduce the inter-particle spacing and therefore reduce the mean free path for phonon transport. This could lead to an increase in the thermal conductivity of the nanocomposite. Conversely, good interfacial adhesion between filler and matrix may form an interfacial layer around the filler. This will increase phonon scattering and hence reduce the thermal conductivity of the nanocomposite. At relatively low rGO contents, a good dispersion is likely to leave the rGO particles isolated, as shown in Fig. 7h. This, coupled with the interfacial layer around the rGO due to strong interfacial adhesion, will increase phonon scattering. Hence, the relatively poor thermal conductivity of the nanocomposite with 0.01 wt% rGO can be understood. As the content of rGO increases, the mean free path length for phonon transport decreases and the rGO particles may begin to form a network as the particles make contact with each other, as observed in the 0.06 wt% rGO nanocomposite (Fig. 8). This may offset the phonon scattering effect due to the interfacial layer, thereby increasing the thermal conductivity of the nanocomposites as observed in the nanocomposites with rGO contents higher than 0.01 wt%. Therefore, the excellent thermal conductivity measured in the nanocomposites can be attributed to the excellent rGO dispersion brought about by the processing technique adopted in this work.

## Conclusions

In this work, a facile, scalable and commercially-viable method has been developed to prepare polymeric nanocomposites of epoxy polymer with very low rGO content, having much improved thermal conductivities. This involves taking advantage of the increased interlayer spacing in GO (compared to that in GNPs) owing to intercalated OCFGs, as well as the compatibility between the OCFGs and the matrix epoxy to achieve a good dispersion of rGO in the nanocomposite via in situ processing. The good dispersion of GO in the aqueous media was transferred to a DGEBA epoxy resin with minimal mechanical energy input. This was followed by in situ reduction of the GO to rGO at 200 °C, which eliminated a substantial amount of the OCFGs.

Electron microscopy revealed a good dispersion of rGO in the nanocomposites, except for the highest content of 0.06 wt% rGO where agglomeration was observed. This was attributed to the tendency of GO to form liquid crystalline structures above certain critical contents. Although addition of rGO led to a decrease in the *T*_g_ and in the resistance of the nanocomposites to thermal oxidation, the storage and tensile moduli were increased significantly. This was attributed to the excellent rGO dispersion and strong interfacial adhesion between the rGO and the epoxy matrix. The presence of defects caused by the low-temperature reduction of the OCFGs led to a reduction in the tensile strength and elongation at break. The observed thermal conductivity of 0.25 W/mK (measured at 30 °C for the nanocomposite with 0.06 wt% of rGO) represents an increase of ~40% compared to the unmodified epoxy. This value is one of the highest thermal conductivity values ever reported for rGO/epoxy nanocomposites having such relatively low contents of rGO. It has been attributed to the excellent dispersion of rGO and large lateral flake size particular to these nanocomposites. These results, taken together, show that it is now possible to tune the properties of an epoxy polymer with a simple and viable method of GO addition.
